# Clinical and Neuroimaging Findings of Sydenham’s Chorea

**Published:** 2014-06

**Authors:** Arzu Ekici, Ayten Yakut, Sevgi Yimenicioglu, Kursat Bora Carman, Suzan Saylısoy

**Affiliations:** 1 Department of Pediatric Neurology; 2 Department of Radiology, Osmangazi University, Faculty of Medicine, Eskisehir, Turkey

**Keywords:** Sydenham’s Chorea; MRI Findings; Chorea Paralytica; Corticosteroids

## Abstract

***Objective:*** Sydenham’s chorea (SC) is thought to be an autoimmune disorder. MRI is generally used to exclude other causes of chorea. There are no typically defined MRI features of SC. In this study we aimed to determine clinical and neuroimaging findings of SC.

***Methods:*** In this study 17 patients with acute SC were retrospectively evaluated. Sydenham’s chorea was diagnosed according to the 1992 revision of the Jones criteria. The other causes of chorea were excluded. Cranial MRI was performed in all patients during the acute phase of SC. Walking, speech and swallowing disorders, muscle weakness, behavioral disorders, treatment, symptom recovery time and recurrence were evaluated.

***Findings*** ***:*** The patients’ mean age was 11.2 years. Behavioral changes, muscle weakness and dysphagia occurred in 70%, 64% and 23% of the patients, respectively. Nonspecific signal hyperintensities were observed in the white matter, brain stem and caudate nucleus in 47% of patients. Two patients who had chorea paralytica were treated successfully with a high dose of intravenous methylprednisolone.

***Conclusion:*** Nonspecific hyperintense white matter abnormalities may be due to the inflammatory process associated with a longer duration of clinical signs. To explain the MRI findings and the pathogenesis of SC, comprehensive studies are needed.

## Introduction

Sydenham’s chorea (SC) is a late manifestation of acute rheumatic fever (ARF) and can occur several months after group A β-hemolytic Streptococcus infections^[^^1^^]^. Although the incidence of ARF and SC has declined significantly in developed countries, they are still serious health concerns in developing countries. Sydenham’s chorea is characterized by involuntary choreiform movements with or without other motor symptoms including facial grimacing, hypotonia, muscle weakness, gait disturbance, difficulty in writing and speaking. Generalized weakness and hypotonia can be so severe that the patient becomes bedridden. This special form of SC is called “chorea paralytica”. Neuropsychiatric symptoms are also commonly observed^[^^2^^]^. 

 There is no specific laboratory test for SC. The diagnosis relies on a careful clinical history and laboratory assessment to rule out other causes of the symptoms, such as systemic lupus erythematosus, drug intoxication, Wilson’s disease, familial chorea and hyperthyroidism^[^^3^^-^^5^^]^. The neuroimaging findings of SC have been rarely reported. In this study, the clinical and cranial MRI findings of 17 patients with SC were evaluated. 

## Subjects and Methods

Seventeen patients with acute SC who were admitted to the Pediatric Neurology Unit of the Eskisehir Osmangazi University Faculty of Medicine were retrospectively evaluated between 2010 and 2012. Sydenham’s chorea was diagnosed according to the 1992 revision of the Jones criteria^[^^1^^]^. Patient medical histories were collected. Physical and neurological examinations were performed. Anti-streptolysin O (ASO), erythrocyte sedimentation rate and the total blood count were routinely obtained from patients at the time of their admission to the hospital. An ASO titer greater than 250 Todd U/mL was considered to be elevated. Tandem MS, urine organic acid analysis and screening for lysosomal enzymes were performed in one patient. Cranial MRI was performed in all patients during the acute phase of SC. MRI examinations were performed on 1.5 T MR scanner (Siemens, VisionPlus, Germany) equipped with the head coil. The MRI protocol included T1 and T2 spin echo sequences in axial planes, fluid attenuation inversion recovery in coronal plane. After 0.1 mmol/kg body weight intravenous gadolinium injection, axial and sagittal T1-weighted image were obtained. The DWI was performed with spin–echo EPI sequence with three b values (0, 500 and 1000).

 The other causes of chorea were excluded. If SC was the only major feature of ARF, an ophthalmic examination, a thyroid function test (TFT), antinuclear antibody (ANA) and anti-dsDNA antibody, serum ceruloplasmin and tests of liver enzymes were performed. 

Chorea was classified as follows:

a)    mild (minimal movements), 

b)    moderate (inconvenient movements for the patient that do not interfere with his or her personal care), 

c)    severe (incapacitating movements requiring the patient to have help for daily activities). 

 Sydenham chorea was also classified according to localization as follows: generalized (movements affecting the whole body) or hemichorea (movements affecting one side of the body). If the patient became bedridden because of generalized hypotonia, they were diagnosed as chorea paralytica^[^^2^^]^. Patients were followed up at one month intervals. Walking, speech and swallowing disorders, muscle weakness, behavioral disorders, treatment, symptom recovery time and recurrence were evaluated. All patients received ARF prophylaxis with benzathine G penicillin. Chorea treatment was also investigated in all patients.

**Table 1:** The clinical findings and treatment of the patients

**Patient** **No**	**Age years/gender**	**Chorea**	**Chorea** **scale**	**Muscle weakness**	**Speech disorder**	**Dysphagia**	**Behavioral changes**	**Carditis**	**treatment**
**1**	14/F	G	3	+	+	-	+	+	VA
**2**	9.5/F	G	3 CP	+	+	+	+	-	VA, HIVMP
**3**	8/F	G	3	+	+	+	+	-	VA
**4**	9.5/M	H	2	-	+	-	-	+	PHB
**5**	11.5/M	H	3	-	+	-	+	-	VA
**6**	11/F	H	2	-	+	-	-	-	VA
**7**	8.5/M	G	3	+	+	+	+	-	VA
**8**	8/F	G	3	+	+	-	+	+	VA
**9**	15/F	G	3	+	+	-	+	-	VA
**10**	6/F	G	3	+	+	-	+	-	VA
**11**	13/M	H	2	-	-	-	-	+	VA
**12**	8/F	H	3	+	+	-	+	-	VA
**13**	12/F	G	3	+	+	-	+	-	VA
**14**	15/F	G	1	-	-	-	-	-	HL
**15**	13.5/F	G	3 CP	+	+	+	+	-	VA, HIVMP
**16**	16.5/F	H	2	-	-	-	-	-	PHB
**17**	12/F	G	3	+	+	-	+	-	VA

G**:** generalized; H: hemichorea; CP: chorea paralytica; VA: valproic acid; PHB: phenobarbital; HL: haloperidol; MP: methylprednisolone; HIVMP: high-dose intravenous methylprednisolone

**Table 2:** The neuroimaging findings of the patients

**Patient** **No**	**MRI finding**	**Control MRI**		**Other findings**
		**Finding**	**Time (months)**	
1	Hyperintensity within the caudate nuclei	Regression	4	-
2	Confluent hyperintense lesions in the bilateral periventricular-subcortical white matter	No change	6 and 12	-
3	Hyperintense foci in white matter on T2-WI	Normal	6	-
4	Hyperintense foci in the white matter on T2-WIs	Normal	6	-
5	Hyperintense foci in the white matter on T2-WIs	No change	6	MR angiography: Normal
6	Hyperintense foci in the white matter on T2-WIs	Normal	6	-
10	Hyperintense foci in the brain stem on T2-WIs	-	-	-
15	A hyperintense cortical lesion in the left temporal region and no contrast enhancement	No change	6 and 12	MR spectroscopy: Lactate peak in the left lateral temporal gyrus

MRI: Magnetic Resonance Imaging

***Findings***

**Fig. 1:**
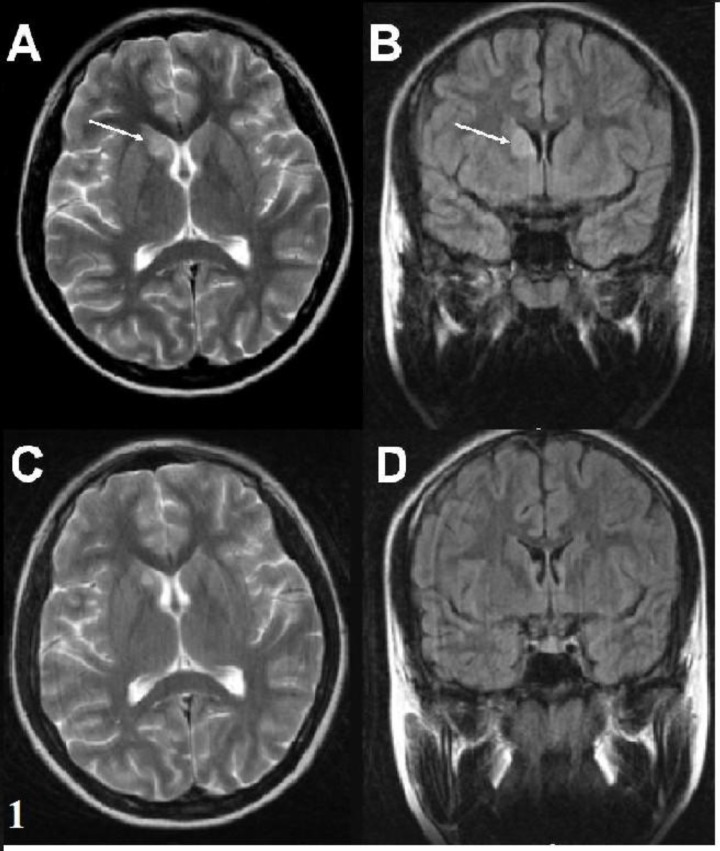
**(** **A,B)** A hyperintense lesion within the right caudate nuclei is observed on both T2-weighted axial image and the FLAIR coronal image. **(C,D)** In the follow-up MRI studies, lesion regression was evident after 4 months (Patient 1).

The patients were predominantly female (13/17). The average age at onset of Sydenham’s chorea was 11.23 years (SD=3.01 years, range 6-16.5 years). Three patients had been previously diagnosed as ARF and the other 14 patients (82%) had a history of throat infection within two months. The serum ASO level was elevated in all patients (ASO >250 Todd U/mL). Sydenham’s chorea was the only major finding of ARF in 76% of the patients and 24% of the patients presented with accompanying carditis. Chorea was mild in 1 (5.9%) patient, moderate in 4 (23.5%) patients and severe in 12 (70.6%) patients. Two (11%) patients were diagnosed with chorea paralytica. Chorea was generalized in 11 (64%) patients and 6 patients had hemichorea (36%). Fourteen (82%) patients had a speech disorder. Behavioral changes, muscle weakness and dysphagia occurred in 70%, 64% and 23% of the patients, respectively. The clinical findings and treatments are summarized in Table 1. Cranial MRI was performed in all patients and abnormalities were observed in 8 (47%) patients. The neuroimaging findings are shown in Table 2. Nonspecific signal hyperintensities were observed on T2-weighted images in the white matter, brain stem and caudate nucleus (Fig. 1). In patient 15, MR spectroscopy demonstrated a hyperintense cortical lesion in the left temporal region, which was suggestive of a dysembryoplastic neuroepithelial tumor (DNET). Control MRI was performed after 4-12 months of follow up in 7 of the patients. Three of 8 patients with neuroimaging findings demonstrated complete resolution after 6 months, while 1 patient’s MRI findings regressed after 4 months. In two patients with chorea paralytica, the hyperintense lesions observed using MRI were unchanged after 12 months of follow up. Fifteen patients were administered valproic acid (VA), phenobarbital (PHB), or haloperidol (HL) treatment for 1-8 months. After the disappearance of the patient's choreiform movements, the medication was tapered over a period of two months. Two patients who had chorea paralytica were administered high-dose intravenous methylprednisolone (HIVMP) (30 mg/kg/day, 5 days). Bedridden and dysphagia improved within the first week and chorea and the other symptoms recovered within 1 month. Choreiform movements of the 15 patients who were not given HIVMP, recovered completely within 1-7 months. Muscle weakness, speech disorders, dysphagia and behavioral changes disappeared in the first and second controls. The mean follow-up period was 9.6 (5-21) months and there was no recurrence during the follow-up period. 

**Fig. 2:**
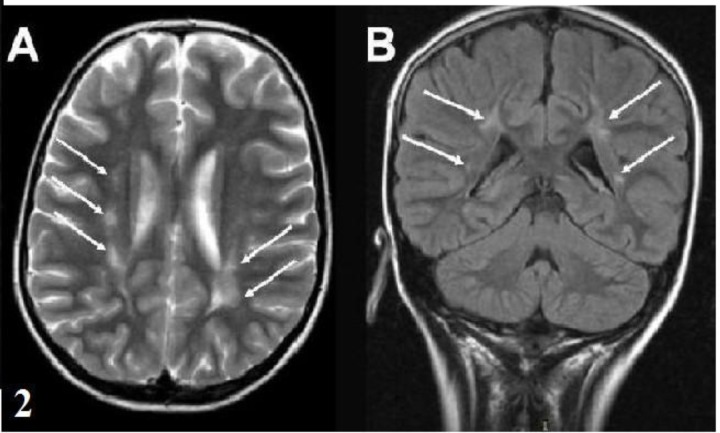
**(A,B)** Both T2-weighted axial image and the FLAIR coronal image show confluent hyperintense lesions in the bilateral periventricular-subcortical white matter. These lesions persist in the follow-up images (Patient 2).

**Fig. 3:**
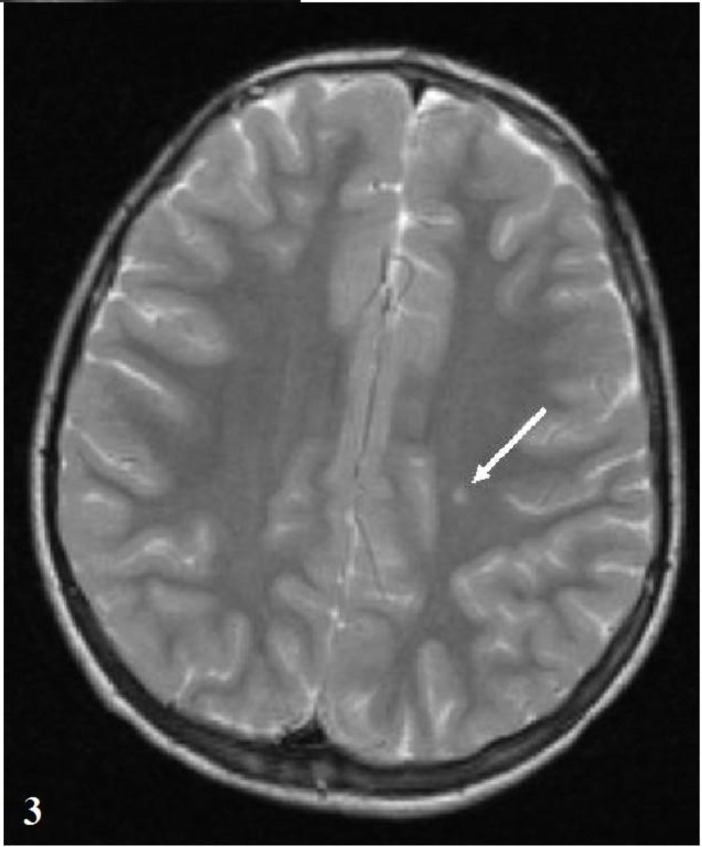
T2-weighted axial image shows a single hyperintense focus in the left frontal white matter (Patient 5).

**Fig. 4:**
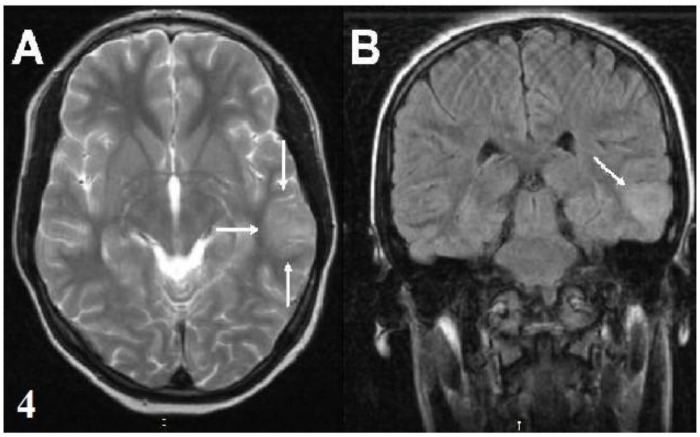
**(A,B)** The hyperintense cortical lesion in the left temporal region on a T2-weighted axial image and a FLAIR coronal image (Patient 15).

## Discussion

Sydenham’s chorea is a poststreptococcal autoimmune disease and it is the most common cause of chorea during childhood^[^^2^^]^. Sydenham’s chorea manifests in children aged 5-15 years, with a female predominance^[^^6^^]^. The major neurological features of SC are involuntary movements, which are exacerbated by stress and disappear during sleep. Chorea is defined as distal, rapid, purposeless movements. They can be generalized or observed on one side of the body (hemichorea). Incoherent speech, dysarthria, muscle weakness and hypotonia are commonly associated features. Hypotonia and weakness have a range of severity from mild to severe. Flaccid quadriparesis, which is also known as “chorea paralytica”, can develop. Neuropsychiatric symptoms, such as emotional lability, crying spells, irritability, tics and obsessive-compulsive signs, are often observed in SC^[^^2^^,^^7^^]^. In this study, 76% of the patients were female and the mean age was 11.2 years. These findings are consistent with the literature^[^^7^^,^^8^^]^. Chorea was generalized in 64% of our patients. More than half of the patients had a speech disorder, behavioral changes and muscle weakness and one fourth of the patients had dysphagia. Our two patients with chorea paralytica had severe weakness and dysphagia, hospitalization was therefore required. 

 Approximately 35% of patients with ARF develop chorea. Cardiac involvement occurs in 42-70.5% of cases with chorea^[^^9^^,^^10^^]^. In this study, SC was the only finding of ARF in 76% of the patients and 24% of the patients presented with accompanying carditis. The serum ASO level was elevated in all patients and 82% of the patients had a history of throat infection^[^^11^^]^. In our study the serum ASO level was elevated in all patients. The ASO titer peaks 3 to 5 weeks after the onset of GABHS pharyngitis and then gradually declines over the following weeks^[^^2^^]^. Although SC is a late complication of GABHS, ASO titers were higher in most patients in some studies. Fusco et al^12^ reported that serum ASO titers were elevated in nine of ten patients with SC. And also elevated ASO was demonstrated in 80% of cases^[^^13^^]^. In the study of Ridel et al^14^, ASO and anti-DNase B titers were elevated in all patients with SC as in our study. It may be speculated that depending on the initial very high titers, ASO titers may still be high at diagnosis of SC.

 Sydenham’s chorea is thought to be an autoimmune disorder, but the exact pathophysiology is still unclear. It is believed that antibodies against group A β-hemolytic Streptococcus (GABHS) cross-react with neurons of the basal ganglia. These anti-basal ganglia antibodies react with the surface of neuronal cells and signal the induction of calcium calmodulin-dependent protein kinase II. Thus, the tyrosine hydroxylase level is elevated and dopamine is released, leading to the movement disorder^[^^15^^,^^16^^]^. Pathological studies have demonstrated neuronal loss, cytoplasmic and nuclear cell changes, gliosis, endothelial swelling, perivascular round cell infiltration and petechial hemorrhages within the cerebral cortex, basal ganglia and thalamus^[^^17^^]^. 

 The diagnosis of SC is difficult without carditis or other manifestations of ARF. Other causes of chorea should be excluded^[^^3^^-^^5^^]^. Although increased ASO titers exist in two thirds of cases, it is not helpful in the diagnosis of SC^[^^18^^]^. Magnetic resonance imaging is generally studied to exclude other causes of chorea. There are no typically defined MRI features in SC. MRI may show varying degrees of signal hyperintensity on T2-weighted images in focal regions, such as the corpus striatum, caudate nucleus, putamen and multiple other areas. These abnormalities may be localized to the basal ganglia, but they are often not consistent with the patient’s clinical signs. The MRI findings of SC typically disappear over time. Therefore, it is thought to develop as a result of vasculitis or inflammation. However, the persistence of these hyperintensities has been reported rarely^[^^8^^,^^19^^-^^21^^]^. In this study, increased signal intensities were detected in the white matter, brain stem and caudate nuclei. Some lesions completely regressed during follow up. We believe that the lesion regression may support an inflammatory origin. In patient 2, the confluent hyperintense lesions on the bilateral periven-tricular-subcortical white matter were still present at the follow-up MRI. These lesions might be secondary to ischemia or demyelination due to vasculitis or inflammation. In patient 15, the hyperintense cortical lesion was most likely consistent with DNET and we considered it to be an incidental finding. In our cases, to interpret all hyperintensities to be related to acute phase of SC may be speculative. However, based on data from other published reports, one or multiple increased signals in the basal ganglia or white matter have been known. In our cases, increased signal intensities were considered suggestive of vasculitis because none of the patients had other previously known causes of vasculitis and our findings were similar to previous reports.

 Sydenham’s chorea is a self-limiting condition with a mean duration of 2-4 months^[^^22^^]^. However, treatment is necessary for patients whose chorea is not mild. Antiepileptics, neuroleptics and phenothiazines have been reported to reduce the abnormal movements by affecting the dopaminergic or alpha-aminobutyric acid (GABA) pathways^[^^2^^,^^23^^,^^24^^]^. Intravenous immunoglobulin, plasma exchange and corticosteroids effectively reduce involuntary movements due to the pathogenesis of autoimmune SC^[^^12^^,^^25^^]^. However, because of the side effects of corticosteroids, their use is recommended only in chorea paralytica^[^^26^^]^. In our study, two patients with chorea paralytica who had MRI findings, persistent choreiform movements of the 15 patients not given HIVMP, received steroids and recovered completely within 1-7 months. Our two patients recovered more quickly with corticosteroids, which is in agreement with the literature^[^^27^^]^. We think that HIVMP appears to be a good choice for bedridden patients with serious dysphagia. Otherwise, classical drug therapy would seem to be more appropriate. In a previous study, recurrence was observed in approximately 30% of patients^[^^28^^]^ and was associated with discontinuation of the antibiotic prophylactic therapy or poor compliance and perhaps with subclinical damage to the basal ganglia following the initial SC episode^[^^29^^,^^30^^]^. There was no recurrence in our study.

## Conclusion

SC might be associated with nonspecific hyperintense white matter abnormalities in MRI. These abnormalities may be due to the inflammatory process associated with a longer duration of clinical signs. Because of the autoimmune role in the pathogenesis of SC, immunomodulatory therapies may be effective. To explain mechanisms behind the MRI findings and the pathogenesis of SC, comprehensive studies are needed.

## Authors’ Contribution

Concept / Design: A. Ekici, A. Yakut, K.B. Carman

Acquisition of Data: A. Ekici, S. Yimenicioğlu, S. Saylısoy 

Data Analysis / Interpretation: A. Ekici, A. Yakut, S. Yimenicioğlu, K.B. Carman, S. Saylısoy 

Manuscript Preparation: A. Ekici, A. Yakut 

Critical Revision of the Manuscript: A. Ekici, S. Saylısoy 

All authors approve final version of the paper.
